# Supplementary material for: Resolving the fine structure in the energy landscapes of repeat proteins
Source: QRB Discov. 2022 Jun 10;3:e7. doi: 10.1017/qrd.2022.4 (PMC10392621; doi:10.1017/qrd.2022.4)
Supplement: Supplementary file 1 [file S2633289222000047sup001.docx]

Supplemental Information - Resolving the Fine Structure in the EnergyLandscapes of Repeat Proteins

Murilo N. Sanches,^†,‡^ R. Gonzalo Parra,^†,fj^ Rafael G. Viegas,^§,‡^ Antonio B. Oliveira Jr.,^ǁ^ Peter G. Wolynes,^ǁ^ Diego U. Ferreiro,^∗,⊥^ and Vitor B. P. Leite^∗,‡^

†*These authors contributed equally to this work*

‡*Department of Physics, S˜ao Paulo State University (UNESP), Institute of Biosciences, Humanities and Exact Sciences, S˜ao Jos´e do Rio Preto, SP, 15054-000, Brazil* fj*European Molecular Biology Laboratory (EMBL), Heidelberg, 69117, Germany*

§*Federal Institute of Education, Science and Technology of S˜ao Paulo (IFSP), Catanduva,*

*SP, 15.808-305, Brazil*

ǁ*Center for Theoretical Biological Physics, Rice University, Houston, TX, USA*

⊥*Instituto de Qu´ımica Biol´ogica de la Facultad de Ciencias Exactas y Naturales, C1428EGA Buenos Aires, Argentina*

E-mail: ferreiro@qb.fcen.uba.ar; [vitor.leite@unesp.br](mailto:vitor.leite@unesp.br)


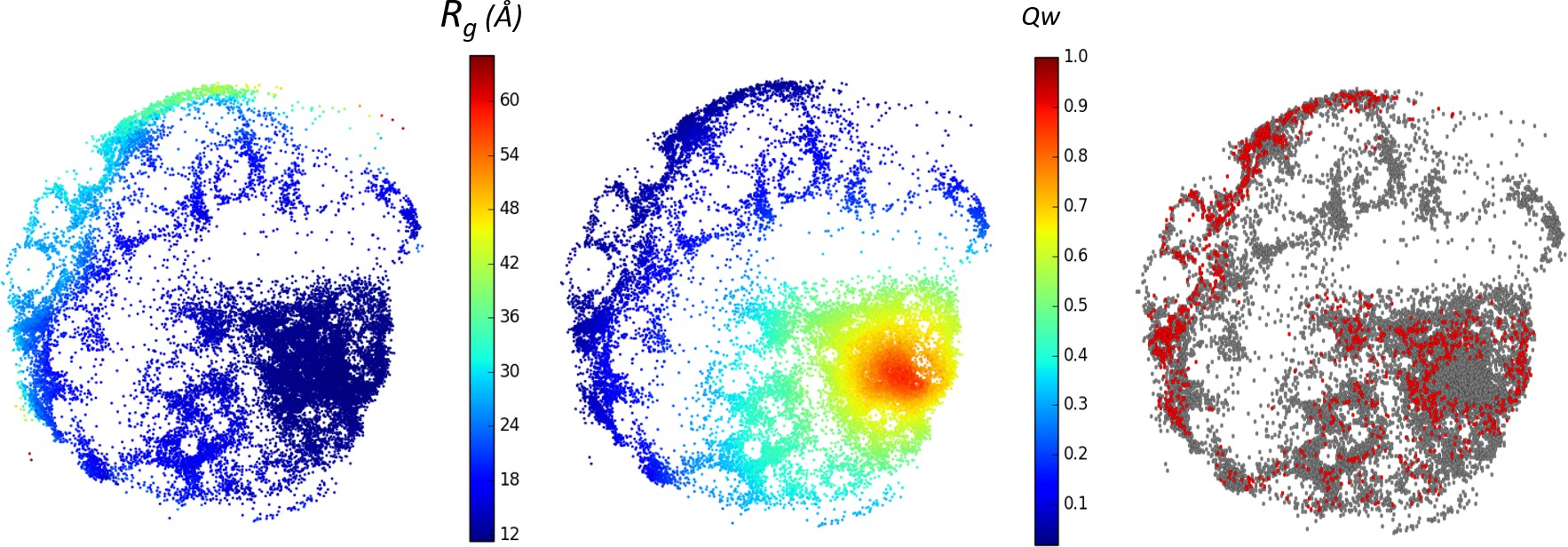


Figure S1: ELViM 2D projection of the conformational phase space of 3ANK as (A) a func- tion of the radius of gyration and (b) a function of *Qw* coordinate. (C) Projection showing the conformations obtained with two different simulations: the unbiased conformations in red, and the biased conformations in grey.


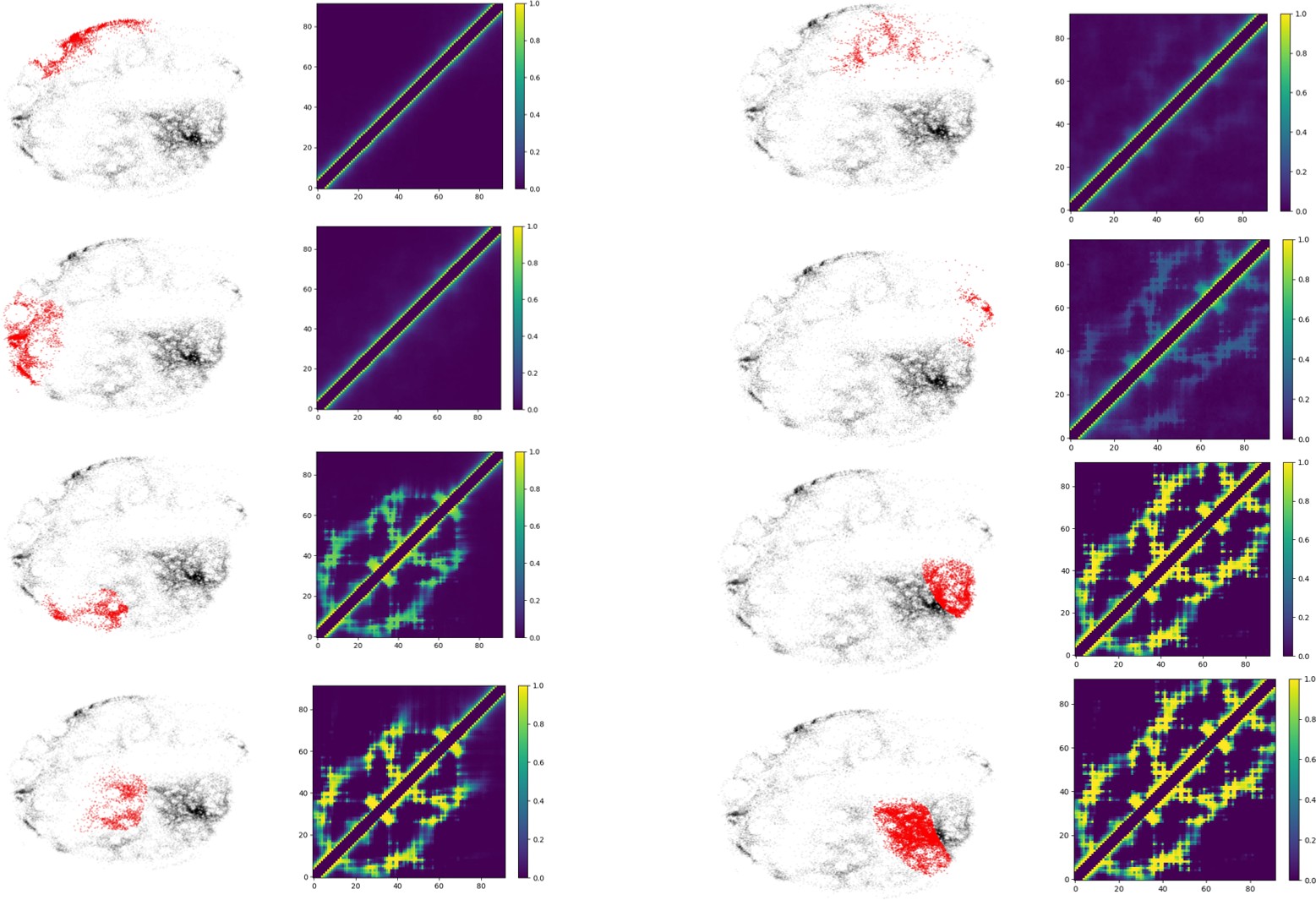


Figure S2: Mean contact maps of the 3ANK structures corresponding to the different regions of the ELViM projection (highlighted in red).


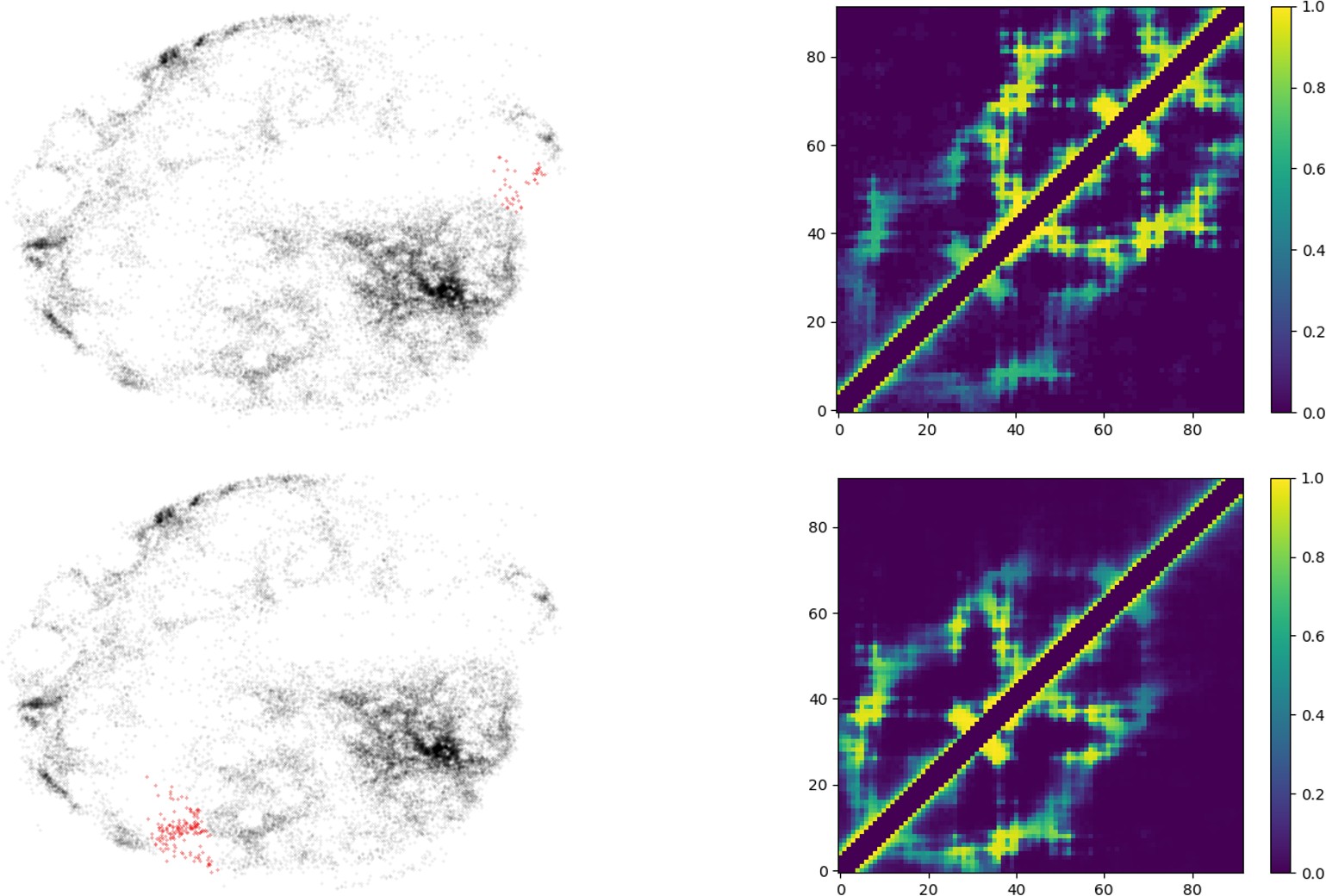


Figure S3: Mean contact maps of the 3ANK transition states with the same values of global *Qo*. The difference between these conformations combined with the fact that they have similar *Qo* values is the reason behind the apparent backtracking.


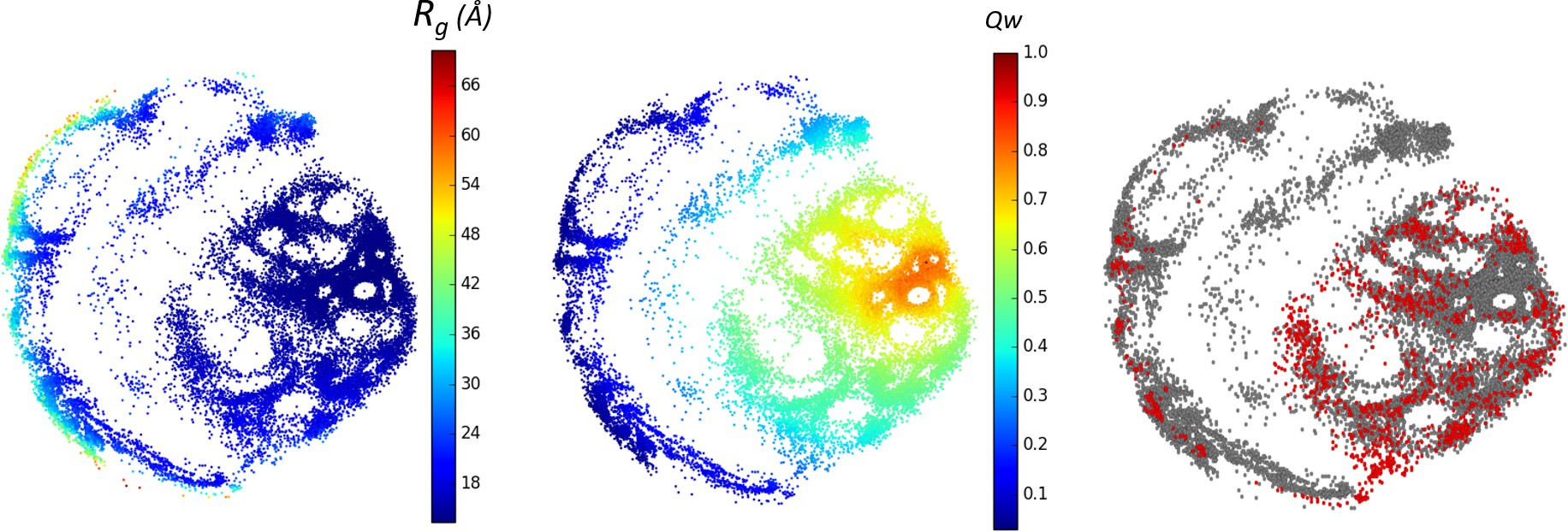


Figure S4: ELViM 2D projection of the conformational phase space of 4ANK as (A) a function of the radius of gyration and (b) a function of *Qw* coordinate. (c) Projection showing the conformations obtained with two different simulations: the unbiased conformations in red, and the biased conformations in grey.


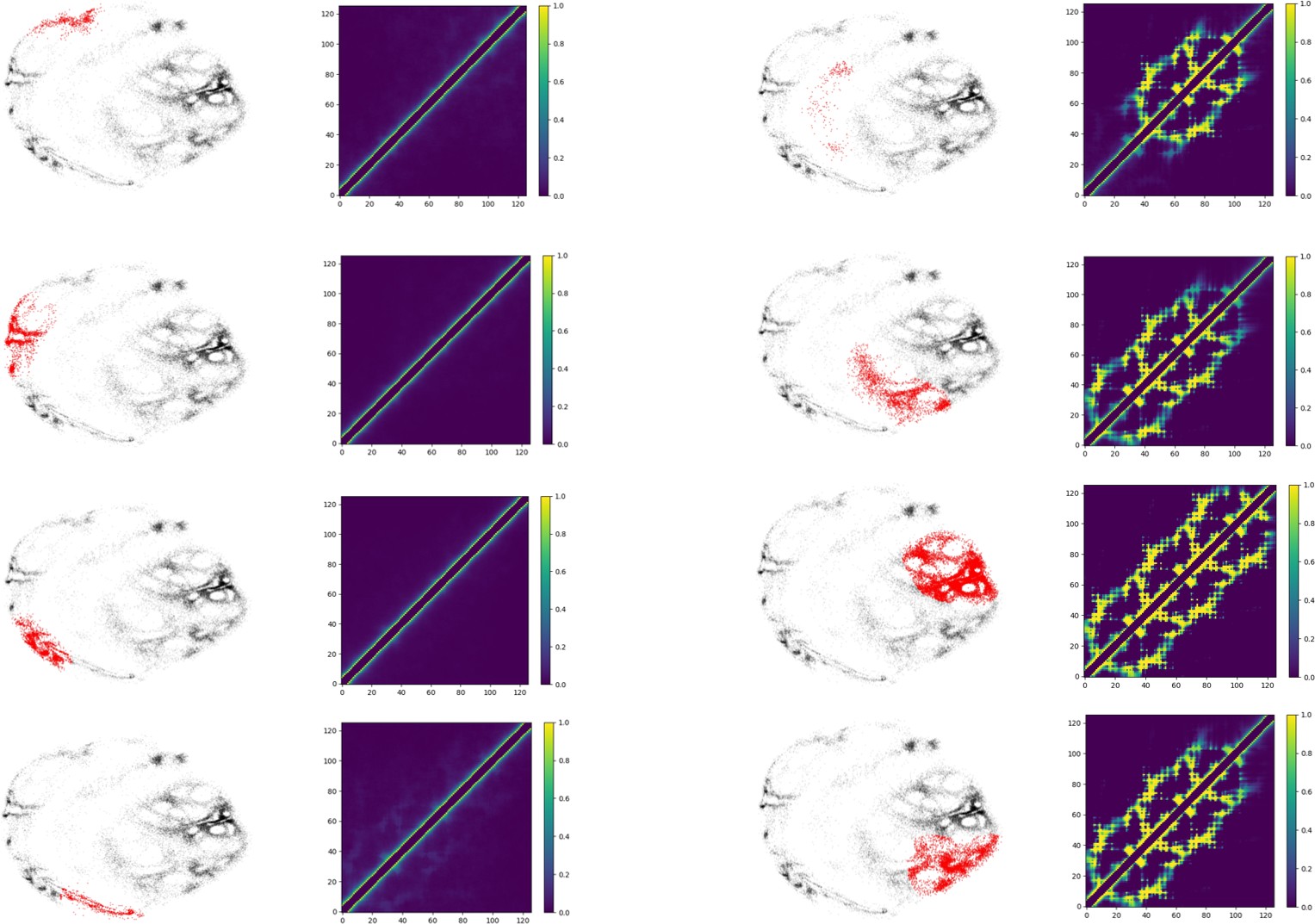


Figure S5: Mean contact maps of the 4ANK structures corresponding to the different regions of the ELViM projection (highlighted in red).


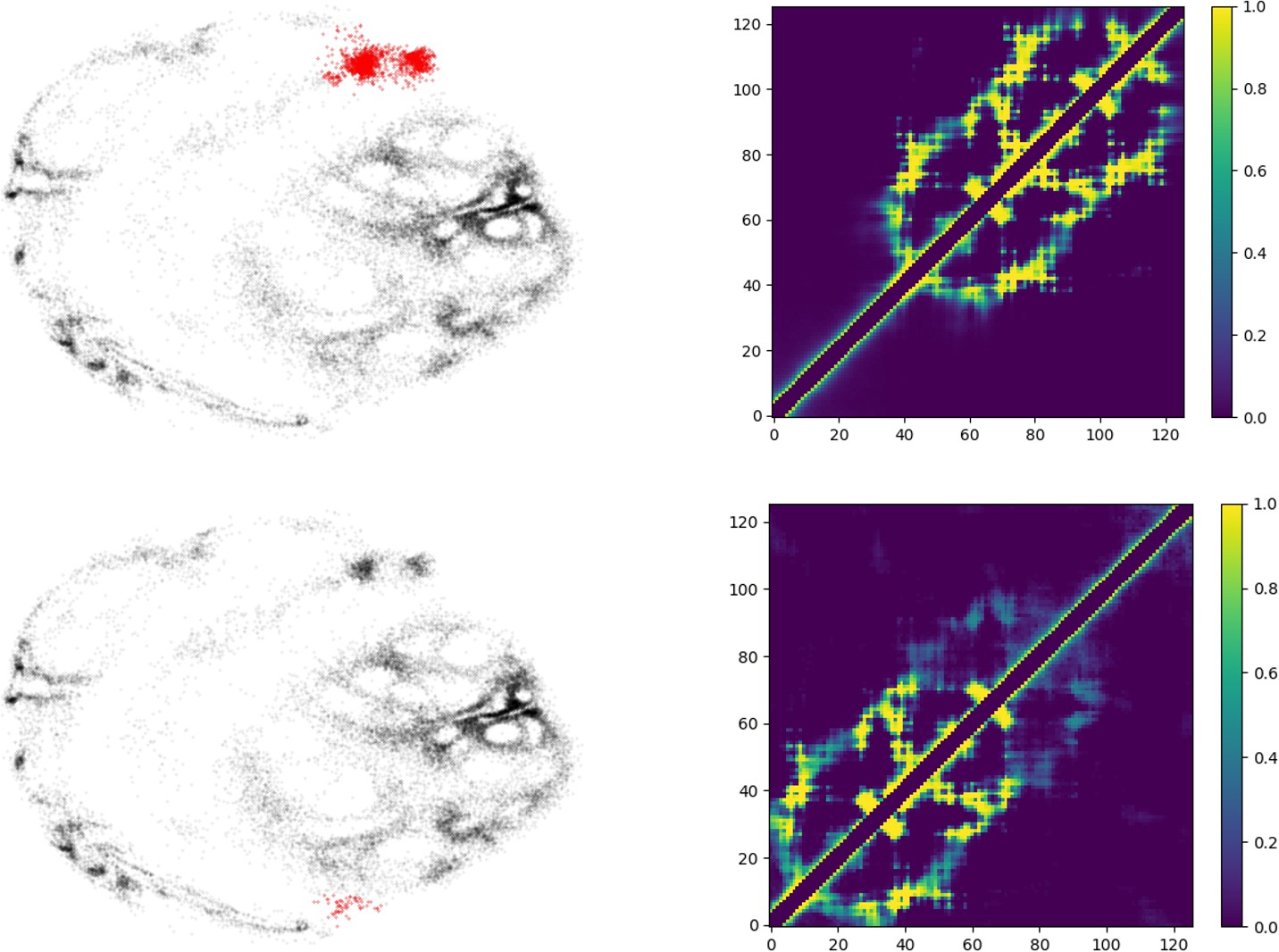


Figure S6: Mean contact maps of the 4ANK transition states with the same values of global *Qo*. The difference between these conformations combined with the fact that they have similar *Qo* values is the reason behind the apparent backtracking.


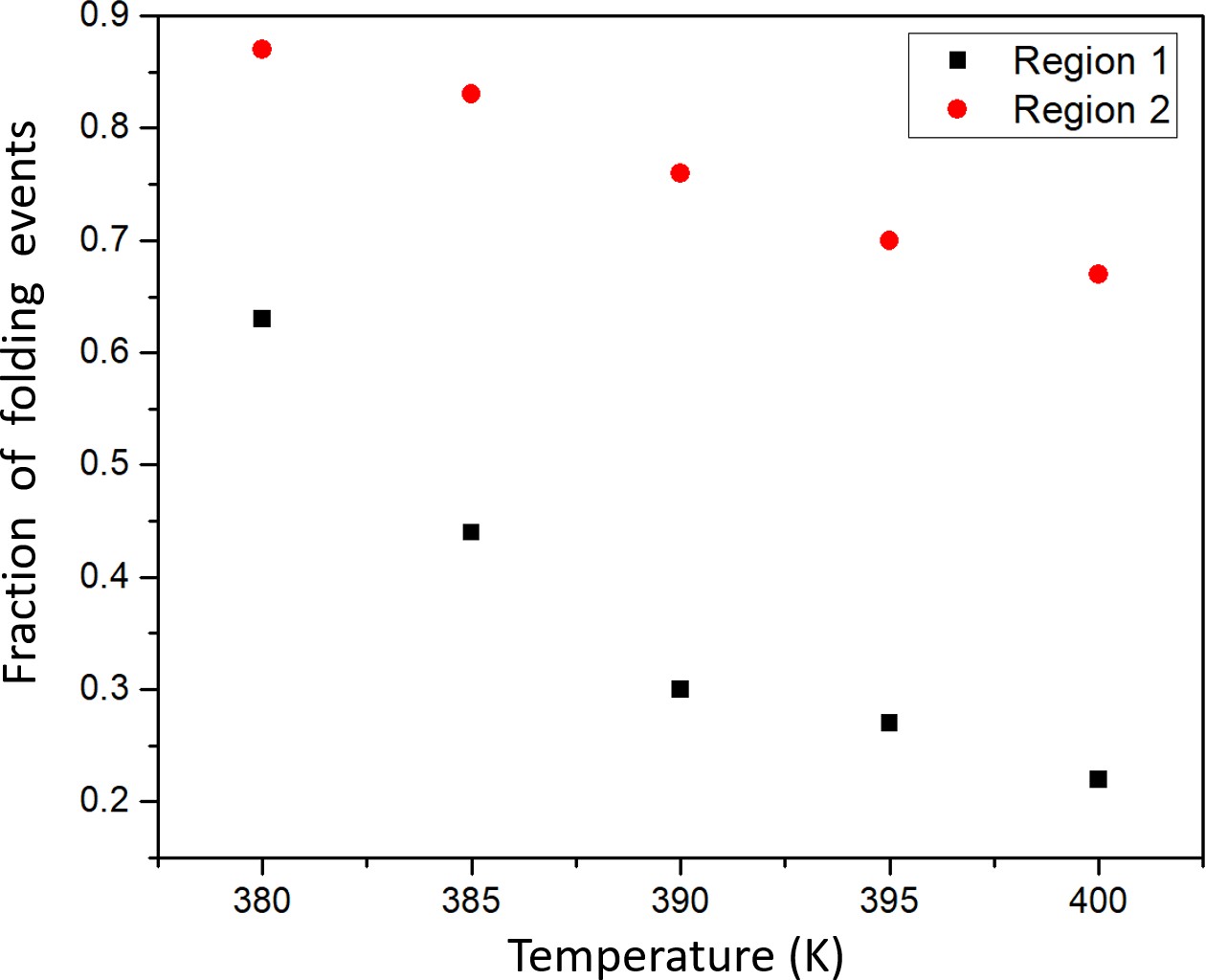


Figure S7: Fraction of folding events for region 1 and region 2 of the 4ANK for each different temperature.


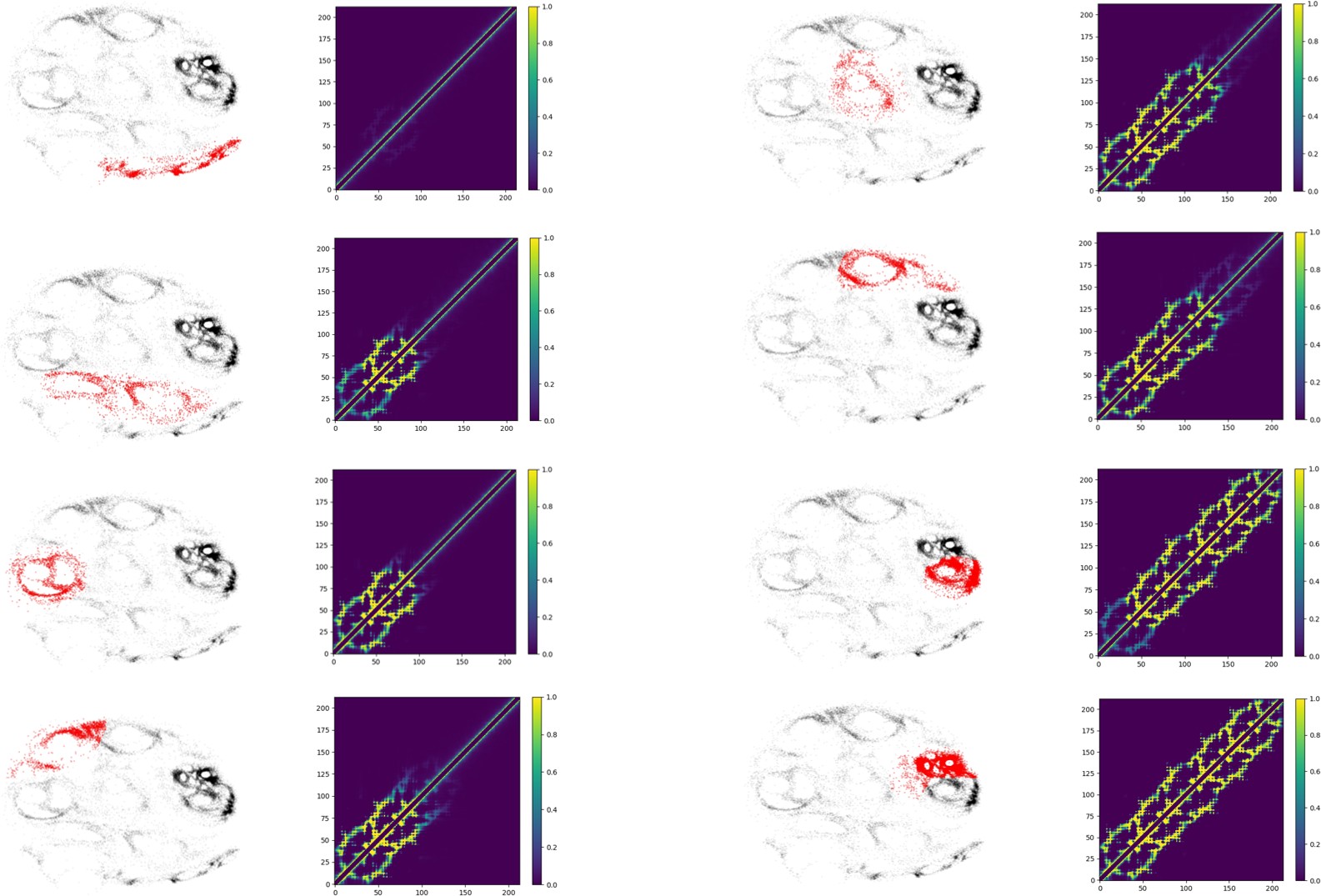


Figure S8: Mean contact maps of the 6ANK structures corresponding to the different regions of the ELViM projection (highlighted in red).


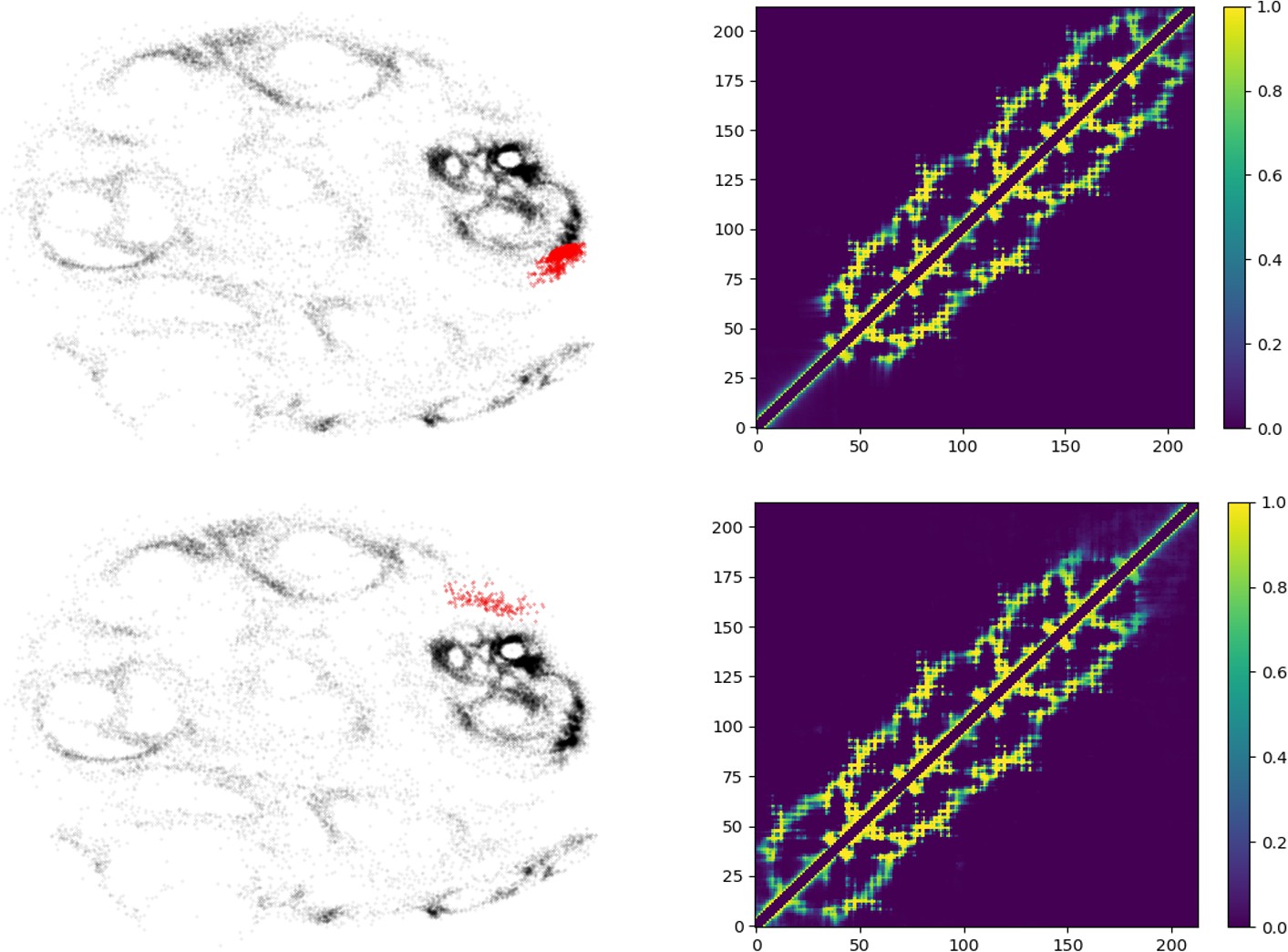


Figure S9: Mean contact maps of the 6ANK transition states with the same values of global *Qo*. The difference between these conformations combined with the fact that they have similar *Qo* values is the reason behind the apparent backtracking.


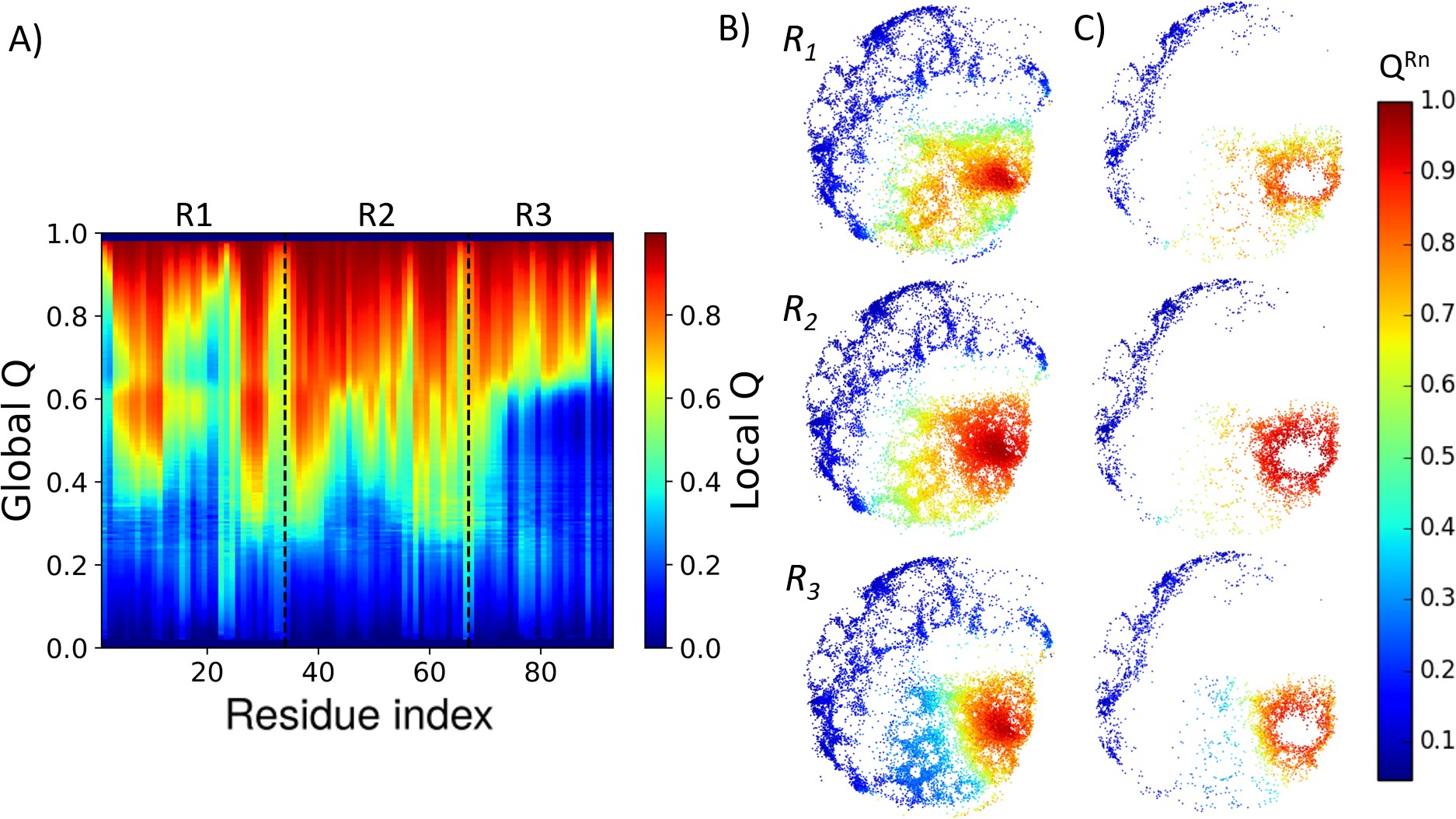


Figure S10: (a) Degree of folding of each residue as a function of *Qo*. The color indicates the average local *Qo^i^* for a specific residue *i* over the entire set of structures of a given global *Qo* for 3ANK. (b) 2-dimensional ELViM projections for 3ANK biased trajectories, as a function of *Qo^Rn^* for each repeat. (c) 2-dimensional ELViM projections for 3ANK unbiased trajectories, as a function of *Qo^Rn^* for each repeat


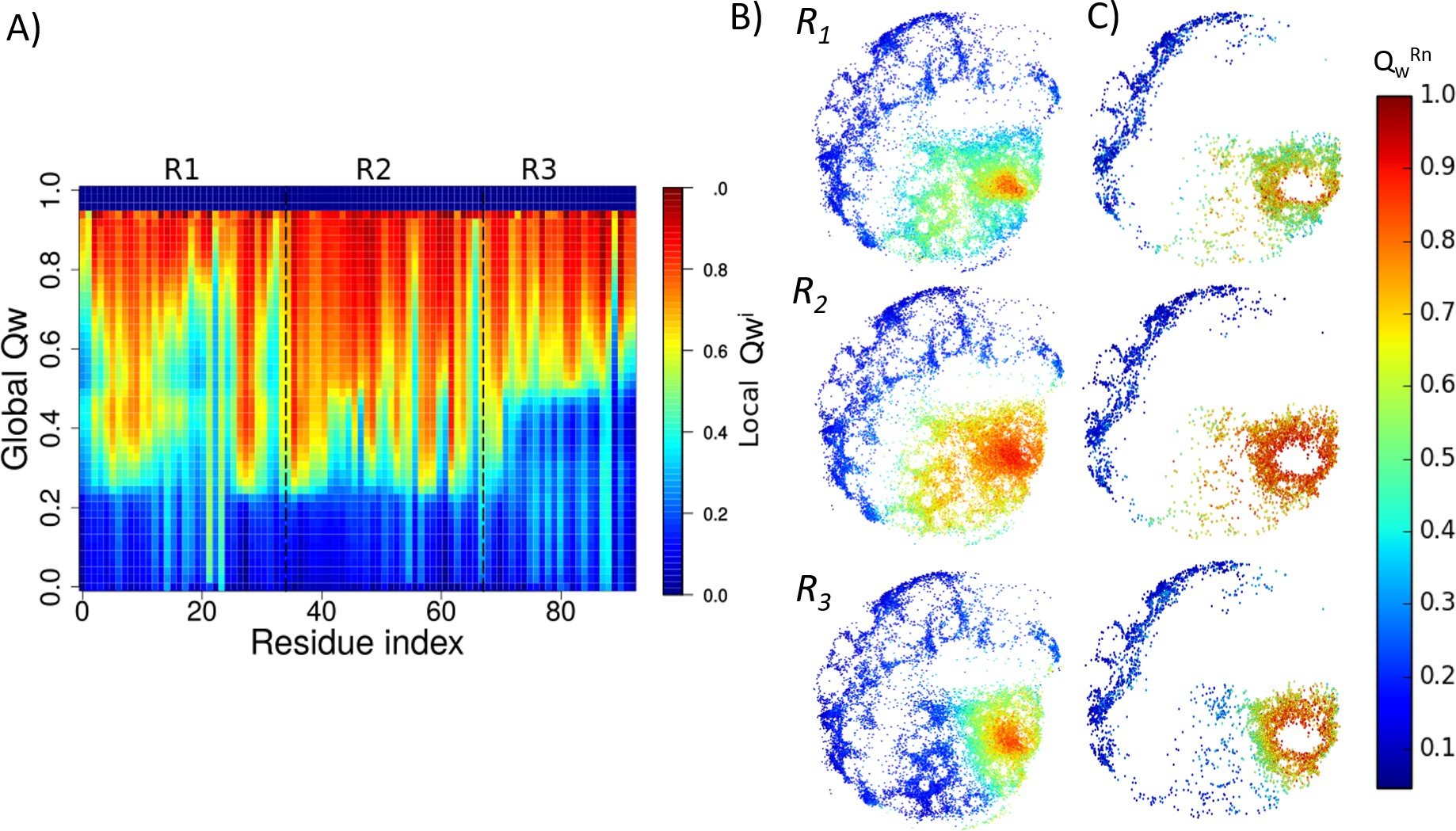


Figure S11: (a) Degree of folding of each residue as a function of *Qw*. The color indicates the average local *Qw^i^* for a specific residue *i* over the entire set of structures of a given global *Qw* for 3ANK. (b) 2-dimensional ELViM projections for 3ANK biased trajectories, as a function of *Qw^Rn^* for each repeat. (c) 2-dimensional ELViM projections for 3ANK unbiased trajectories, as a function of *Qw^Rn^* for each repeat.


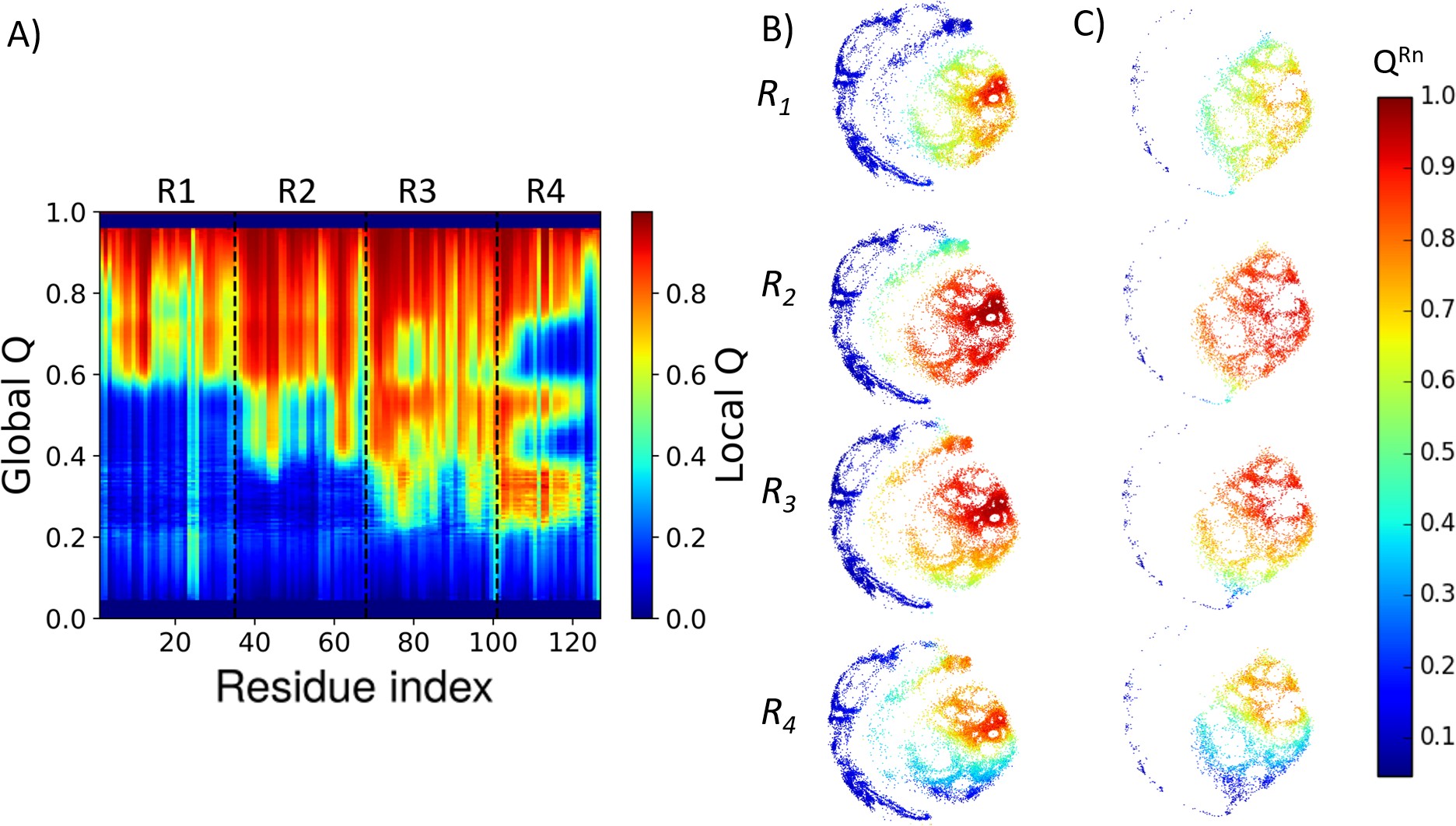


Figure S12: Analyses of the 4ANK repeats formation. (a) Degree of folding of each residue as a function of *Qo*. The color indicates the average local *Qo^i^* for a specific residue *i* over the entire set of structures of a given global *Qo* for 4ANK. (b) 2-dimensional ELViM projections for 4ANK biased trajectories, as a function of *Qo^Rn^* for each repeat. (c) 2-dimensional ELViM projections for 4ANK unbiased trajectories, as a function of *Qo^Rn^* for each repeat.


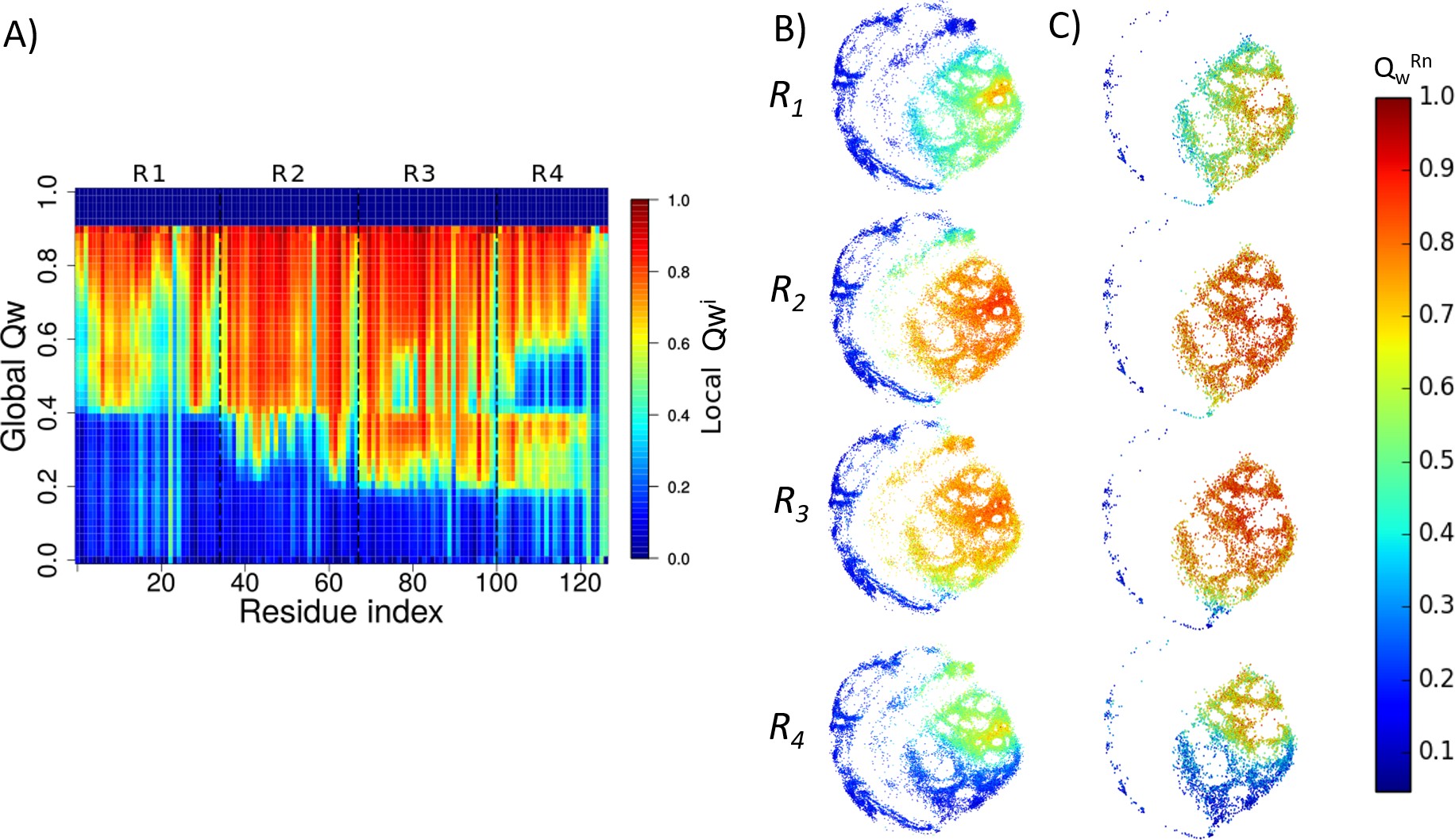


Figure S13: Analyses of the 4ANK repeats formation. (a) Degree of folding of each residue as a function of *Qw*. The color indicates the average local *Qw^i^* for a specific residue *i* over the entire set of structures of a given global *Qw* for 4ANK. (b) 2-dimensional ELViM projections for 4ANK biased trajectories, as a function of *Qw^Rn^* for each repeat. (c) 2- dimensional ELViM projections for 4ANK unbiased trajectories, as a function of *Qw^Rn^* for each repeat


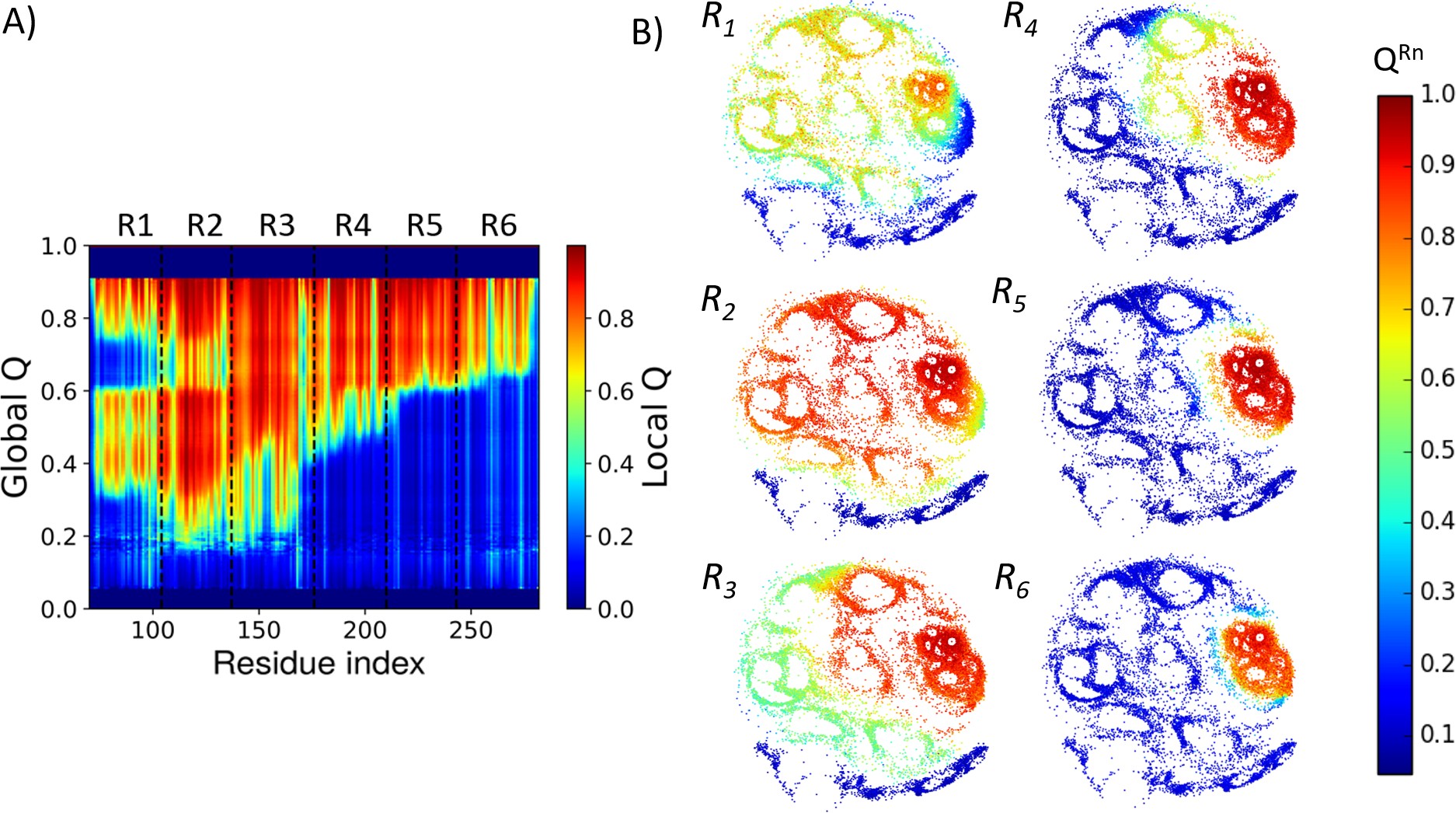


Figure S14: (a) Degree of folding of each residue as a function of *Qo*. The color indicates the average local *Qo^i^* for a specific residue *i* over the entire set of structures of a given global *Qo* for 6ANK. (b) 2-dimensional ELViM projections of 6ANK biased trajectories, as a function of *Qo^Rn^* for each repeat.


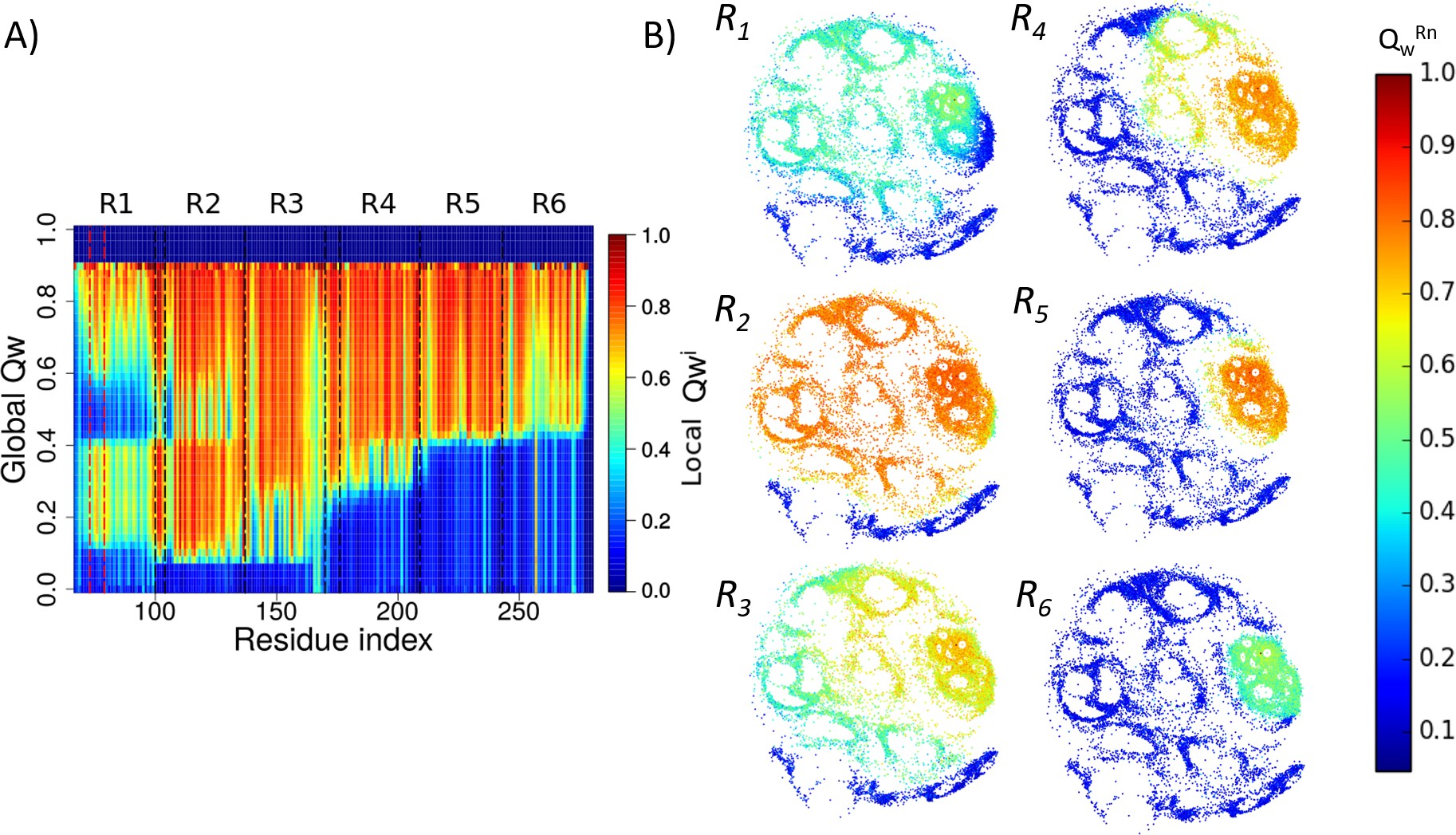


Figure S15: (a) Degree of folding of each residue as a function of *Qw*. The color indicates the average local *Qw^i^* for a specific residue *i* over the entire set of structures of a given global *Qw* for 6ANK. (b) 2-dimensional ELViM projections of 6ANK biased trajectories, as a function of *Qw^Rn^* for each repeat.
